# Textural and physical properties of breast fillets with myopathies (wooden breast, white striping, spaghetti meat) in Canadian fast-growing broiler chickens

**DOI:** 10.1016/j.psj.2022.102309

**Published:** 2022-11-05

**Authors:** Chaoyue Wang, Sunoh Che, Leonardo Susta, Shai Barbut

**Affiliations:** ⁎ Department of Food Science, Ontario Agricultural College, University of Guelph, Guelph, Ontario, Canada N1G 2W1; † Department of Pathobiology, Ontario Veterinary College, University of Guelph, Guelph, Ontario, Canada N1G 2W1; ‡ Adaptation Physiology Department, Wageningen University, The Netherlands

**Keywords:** meat quality, poultry myopathies, spaghetti meat, texture, wooden breast

## Abstract

The combined effects of different severities of Wooden Breast (**WB**), White Striping (**WS**), and Spaghetti Meat (**SM**) were examined in 300 chicken breast fillets from 10 flocks. Severity (0 = absent, 1 = mild, noticeable upon close inspection, 2 = severe), noticeably altered from normal breast fillet (**NB**). Results showed that any combination of myopathies and severity resulted in significantly elevated compression force, pH and peak counts measured by the shear force test. With the exception of mild WB + mild WS, all combinations resulted in significantly higher drip loss, cooking loss and lightness value. Overall, the quality of fillets was affected the least by WS, while negatively affected the most by SM. There were limited effects on fillet quality from mild WB but major deleterious effects from severe WB.

## INTRODUCTION

The poultry industry is one of the largest meat industries in the world with an estimated annual production of around 27 billion kg in North America alone (National Chicken Council, 2020). The demand for poultry products is estimated to increase by 120% by 2050 as compared to beef and pork, which are expected to increase by 66% and 43%, respectively (Mottet and Tempio, 2017). To help meet demand, the growth rate and market weight for broilers has increased by 100% over the past 50 yr (Zuidhof et al., 2014; Barbut, 2015). Despite the advancement in faster-growing broiler production, many new challenges have emerged in recent years (Sihvo et al., 2017). An important one is to reducing quality defects caused by myopathies, which have resulted in >$ 1billion annual losses to the North American poultry industry (Barbut, 2019).

Myopathies such as Wooden Breast (**WB**) and White Stripping (**WS**) were first documented about a decade ago. Morphologically, these conditions are characterized by accumulation of fibrous tissue and fat with inflammatory infiltrates (of variable severity) in the affected fillets (Petracci et al., 2019). These changes impact physical parameters of the breast meat, affecting texture, water holding capacity (drip and cooking losses), pH, and color (Lorenzi et al., 2014; Chatterjee et al., 2016; Dalle Zotte et al., 2017; Malila et al., 2018; Petracci et al., 2019). A new myopathy called Spaghetti Meat (**SM**), has recently emerged and is currently an important cause for quality defects in chicken breast fillets (Soglia et al., 2019; Barbut, 2019). Although some studies have been conducted on the properties and causes of SM, the amount of knowledge and data are limited compared to WB and WS (Petracci et al., 2019). Furthermore, WB, WS, and SM are not mutually exclusive and often can be present together within a given breast fillet (Baldi et al., 2019). To date, very few reports have documented the combined effects of these myopathies on meat quality. Therefore, the goal of our study was to examine how the combination of these three myopathies affects the physical properties of chicken breast fillets obtained from a Canadian large commercial processing plant.

Changes in tissue composition, due to myopathies, influence parameters such as pH, color, drip loss, and compression force of the raw meat (Mudalal et al., 2015; Dalle Zotte et al., 2017; Tasoniero et al., 2020). Cooking loss and shear force tests are commonly used to assess quality of cooked fillets (Zhuang and Savage, 2013; Baldi et al., 2018). Those parameters were therefore selected in this study to compare the effects of combined myopathies. The Meullenet-Owens Razor shear method was used as the shear force test, because it does not require pre-sample cutting, and testing is performed directly on cooked fillets (Cavitt et al., 2005; Morey and Owens, 2017). Peak counts from the shear force test were also determined as Bowker and Zhuang (2019) suggested that they could be an indicator of myopathies.

## MATERIALS AND METHODS

### Sample Collection, Categorization, Color, and pH

A total of 300 boneless/skinless chicken breast fillets, specifically selected for their combination of myopathies, were collected from the deboning line of a large Ontario, air-chilled poultry processing plant during 10 different visits stretching over 6 mo. The plant used electric stunning and the exsanguination to deboning time was 3 h. The birds were mixed-sex Cobb500 (36 d, 2.4 kg) and mixed-sex Ross708 (40 d, 2.4 kg). Each time, 30 fillets with a combination of myopathy traits were individually selected, packaged, and transported, on ice, to the University within 1 h of collection. Each sample was categorized based on the presence and severity of WB (assessed by hand palpation), WS (visual inspection of white striations parallel to the orientation of the muscle fibers), and SM (visual inspection of loose or detached muscle fibers). Scores employed were: 0 (normal breast, NB); 1 (mild, noticeable upon close inspection); and 2 (severe, noticeably altered from NB). For SM, fillets with a score of 0 and 1 were selected, since SM disturbs the fillet's surface and renders several tests impossible or highly inaccurate. The scale used largely follows Baldi et al. (2018) and Che et al. (2022) where SM is evaluated based on presence or absence of the myopathy. A score of 0 is a normal fillet, and 1 indicates presence of loose and detached muscle fibers on the cranial region of the fillet based on visual inspection. Overall, the rating system is comparable to previous studies (Kuttapan et al., 2016; Tijare et al., 2016). Note, a severity rating of 3 used in some previous studies, was not used here as such samples (from average broiler weight of 2.3 kg) could not be clearly classified as reported for 4.0 kg broilers investigated by others. pH and color measurements were obtained from the cranial part of fillets, within 5 h postmortem, using a pH meter (HI 98163, Hanna Instruments, Woonsocket, RI) and a color meter (Pro Color Sensor, Nix Sensor Ltd., Hamilton, ON). Parameters measured included: lightness (L*), redness (a*), yellowness (b*), chroma (C), and Hue (H) under the light source employed by the meter (D50 illuminant). Data were later converted to D65 illuminant, to allow comparison with this more common illuminant used in other scientific reports. Breast fillets were then placed into labeled bags and stored at 4°C. The overall number of samples collected for each group was: 81 (NB), 38 (WB1 WS1), 32 (WB1 WS1 SM1), 33 (WB2 WS1), 69 (WB2 WS1 SM1), 11 (WB2 WS2), 36 (WB2 WS2 SM1).

### Drip Loss

The difference in the weight of the collected and stored breast fillets (<5 h postmortem and 24 h storage in plastic bags at 4°C) was determined. Drip loss was calculated as the percentage of the weight difference.

### Compression Force

Each raw fillet was compressed 3 times (each compression was >1 cm apart from the previous) on the cranial region of the fillet to 80% of their original height, using a texture analyzer (TA.XT Plus Texture Analyzer, Stable Micro Systems, Godalming, Surrey, UK), employing a 10 mm diameter flat probe. The load cell capacity was 30 kg, trigger force 1N, the test speed 5 mm/s, as described by Sun et al. (2018). The compression value (in Newtons) was recorded as the maximum force at 80% of the initial height of the fillet (compressed for 20%, based on the initial height). The method followed a myopathy study by Soglia et al. (2019).

### Sample Preparation and Cooking Loss

Breast fillets were packed in vacuum bags after the compression test. A water bath (Haake, model W26, Dieselstr, Germany) set to 78°C was used for cooking to an internal temperature of 72°C; measured by a thermocouple. This temperature is a common practice used in Canada and is based on our industry partner's suggestion. The cooked fillets were cooled and stored at 4°C for 24 h. Cooking loss, measured the next day, and expressed as the percentage of the weight difference between the weights of cooked and raw samples.

### Instrumental Texture Analysis

Shear force and peak count were measured using the texture analyzer, with the so-called blunt MORS blade which is 0.5 mm thick, 8.9 mm wide, and 30 mm high (Meullenet et al., 2004). Similar to the established method (Meullenet et al., 2004; Lee et al., 2008; Morey and Owens, 2017), fillets were sheared 6 times (1cm distance between each shear test) perpendicular to the muscle fibers, on the ventral surface of the cranial part of the cooked fillet. Samples were sheared to a depth of 20 mm at a crosshead speed of 10 mm/s with a trigger force set at 0.1 N and peak numbers reported.

### Statistical Analysis

Statistical software package (SAS 9.4, SAS Institute Inc., Cary, NC) was used to perform analysis of variance. The GLM procedure was used with myopathy combination as the main effect to determine the influence of combined myopathies on drip loss, pH, color, compression force, cooking loss, and shear peak count. One-way ANOVA and Tukey multiple comparison analysis was performed to separate the means (*P* < 0.05).

## RESULTS AND DISCUSSION

A total of 6 myopathy combinations and one normal breast were categorized during our sampling. Several categories were excluded from the analysis as they were very low in numbers or not found during sampling (categories excluded from this study were WB1 WS2, WB1 WS2 SM1, and WB2 WS2).

### Effects of Myopathies on Raw Fillets

The effect of myopathies on the pH of breast fillets has been extensively studied (Kuttapan et al., 2017; Tasoniero et al., 2020), reporting that pH increases when myopathies are present. Our results also show that pH was greater (5.83–6.04) in all breast fillets when a myopathy combinations were present (except the WB1 WS1 SM1) vs. the pH found in NB (5.71; Table 1). A similar pH range was reported by Baldi et al. (2019) for NB vs. samples with myopathies.

**Table 1:** Effects of combination of broiler breast fillet myopathies (NB = Normal Breast; WB = Wooden Breast; WS = White Stripping; SM = Spaghetti Meat) and different severities of myopathies (0 = absent, 1 = mild, 2 = severe) for different quality parameters on raw fillets.

	Colorimetry					
Myopathies	L*	a*	b*	Chroma	Hue	pH
NB	38.50 ± 0.27^c^	0.71 ± 0.09^a^	3.18 ± 0.18^c^	3.42 ± 0.16^c^	80.52 ± 4.95^a^	5.71 ± 0.02^d^
WB1 WS1	38.87 ± 0.35^c^	0.74 ± 0.17^a^	4.22 0± 0.32^bc^	4.44 ± 0.31^bc^	76.68 ± 2.63^a^	5.83 ± 0.02^bc^
WB1 WS1 SM1	40.65 ± 0.39^b^	1.29 ± 0.29^a^	4.31 ± 0.36abc	4.77 ± 0.35^ab^	71.13 ± 4.14^a^	5.74 ± 0.02^c^^d^
WB2 WS1	41.98 ± 0.29^ab^	1.00 ± 0.12^a^	4.85 ± 0.22^ab^	5.08 ± 0.21^ab^	76.67 ± 1.72^a^	5.89 ± 0.02^b^
WB2 WS1 SM1	41.73 ± 0.26^ab^	1.07 ± 0.18^a^	4.52 ± 0.20^ab^	4.86 ± 0.21^ab^	76.18 ± 1.91^a^	5.90 ± 0.02^b^
WB2 WS2*	42.74 ± 0.44^a^	1.22 ± 0.27^a^	4.87 ± 0.51^ab^	5.11 ± 0.51^ab^	76.07 ± 3.25^a^	6.04 ± 0.03^a^
WB2 WS2 SM1	41.78 ± 0.36^ab^	1.10 ± 0.19^a^	5.62 ± 0.30^a^	5.85 ± 0.29^a^	77.65 ± 2.28^a^	5.88 ± 0.02^b^

a-d Means (N: NB = 78. WB1 WS1 = 40. WB1 WS1 SM1 = 28. WB2 WS1 = 73. WB2 WS1 SM1 = 69. WB2 WS2 = 11. WB2 WS2 SM1 = 36) ± SE followed by different superscript in a given column are significantly different (*P* < 0.05).

⁎ WB2 WS2 data was not used in the discussion due to low sample count.

Different opinions have been presented regarding effects of myopathies on the color of breast fillets (Petracci, 2019). Some reported that lightness (L*) value of fillets may be a good indicator of WS or WB (Alnahhas et al., 2016; Cai et al., 2018). However, others suggested that it could not be used to differentiate the presence or severity of myopathies (Mudalal et al., 2015; Bowker and Zhuang, 2016; Baldi et al., 2018). Our results show that myopathy combinations (except the WB1 WS1) resulted in higher L* values compared to NB samples (Table 1). However, the L* value could not be used to differentiate between different types of myopathies, or combinations thereof. Several studies suggested redness (a*) values to be an effective indicator for the presence of myopathies (Petracci et al., 2013; Dalle Zotte et al., 2017), while others reported it to be ineffective (Mudalal et al., 2015; Bowker and Zhuang, 2016; Baldi et al., 2018). Our results are inconclusive about the use of a*, partly because there were some variations among samples, resulting in high standard errors. Our results reveal that yellowness (b*) was higher in breast fillets with myopathies when WB2 is present (Table 1). This is in agreement with several other studies (Dalle Zotte et al., 2017; Kuttappan et al., 2017). While previous studies have generally not reported chroma and hue data, our study show higher chroma when most myopathy combinations were present (Table 1). This was also observed by Dalle Zotte et al. (2017). However, our hue values were not affected by the presence of myopathies, unlike the study by Dalle Zotte et al. (2017).

**Figure 1:**
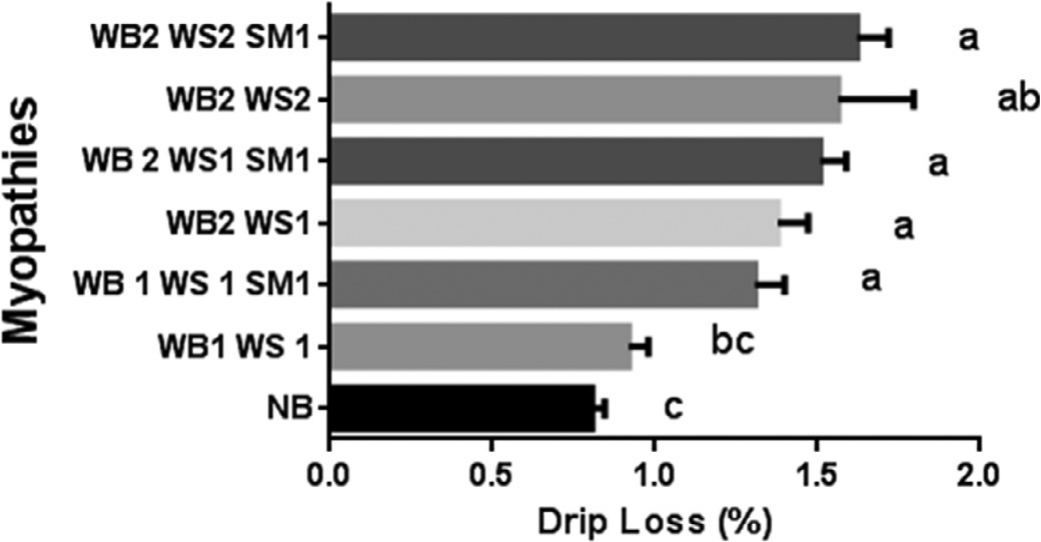
Drip loss (%) for raw Normal Breast (NB) fillets, and fillets with combination of different types of myopathies: Wooden Breast (WB); White Stripping (WS); Spaghetti Meat (SM), and severity: (0 = absent; 1 = mild; 2 = severe). Groups with different letters are significantly different (*P* < 0.05). Bars show standard error.

Our findings show that all myopathy combinations, except WB1 WS1, resulted in significantly higher drip loss of the raw fillets (Figure 1). While severe White Stripping (WS2) or Wooden Breast (WB2) significantly increased drip loss in other studies, a mild degree of myopathies such as WS1 or WB1 did not significantly affect drip loss (Alnahhas et al., 2016; Dalgaard et al., 2018). Our study found that mild Spaghetti Meat (SM1) alone is sufficient to significantly increase drip loss. Baldi et al., (2018) and Tasoniero et al. (2020) also observed a significant increase in drip loss due to SM. In the current study, drip loss was significantly higher compared to control in fillets with any combination of myopathies with the exception of WB1 WS1 (Figure 1). A drip loss value of 1.62% was found in the combination of the most severe myopathies (WB2 WS2 SM1), which was double as the drip loss from normal fillets (0.81%).

**Figure 2:**
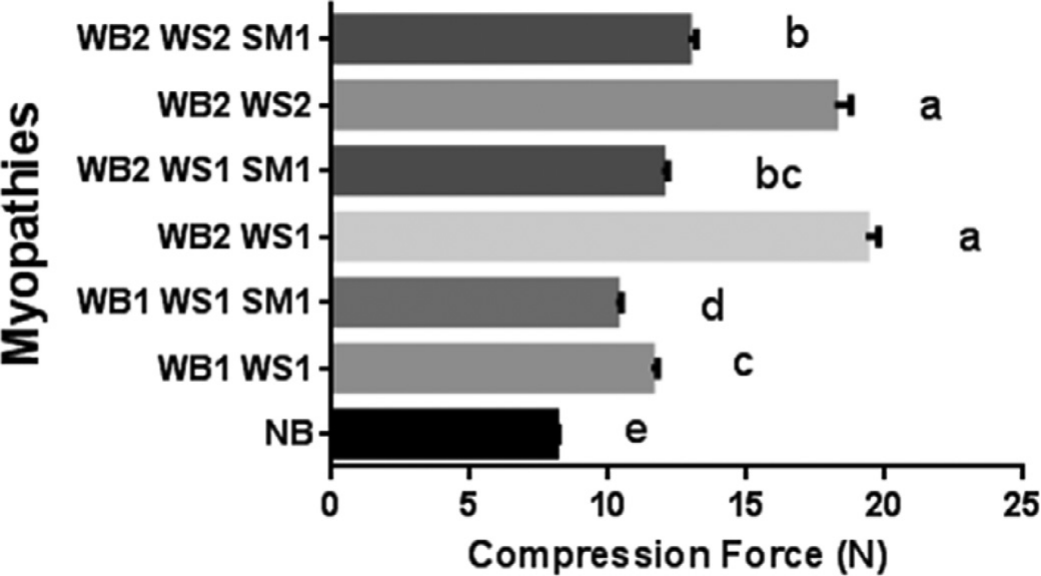
Compression force for raw Normal Breast (NB) fillets, and fillets with a combination of different types of myopathies: Wooden Breast (WB); White Striping (WS); Spaghetti Meat (SM), and severity: (0 = absent; 1 = mild; 2 = severe). Groups with different letters are significantly different (*P* < 0.05). Bars show standard error.

The presence of WB, of any severity, resulted in greater (*P* < 0.05) compression force of the raw breast fillets compared to NB (Figure 2). This is in agreement with previous studies (Dalgaard et al., 2018; Sun et al., 2018). A combination of WB and WS resulted in lower compression force compared to only WB, which differs from inconclusive reports from other studies (Mudalal et al., 2015). Our results also show that the presence of SM with any myopathy combinations resulted in lower compression force compared to fillets with comparable severity of WB and WS, but without SM. Although lower compression force translates to softer fillets, visual alteration from SM, lowered water holding capacity and other functionalities may lead to rejection of the product by consumers, despite no indication of food safety hazard (Petracci et al., 2019).

### Effects of Myopathies on Cooked Fillets

Previous studies point out that the severity of myopathies can affect cooking loss (Kuttapan et al., 2016; Muldalal., 2016). The present study also shows that, compared to normal fillets, cooking loss increases for all myopathy combinations except WB1 WS1 (Table 2). This finding differs from 2 reports in which mild Wooden Breast (WB1) was associated with increased cooking loss (Dalgaard et al., 2018; Tasoniero et al., 2019). In any case, differences in bird age, weight, chilling method (at the processing plant), and cooking procedure are possible causes for this discrepancy. The presence of SM significantly increased cooking loss compared to WB1 WS1 breast fillets. However, SM did not significantly affect cooking losses for WB2 breast fillets. Also, the severity of WS did affect cooking loss, and this is in agreement with some studies (Alnahhas et al., 2016; Bowker and Zhuang, 2016).

**Table 2:** Effects of the combination of broiler breast fillet myopathies (NB = Normal Breast; WB = Wooden Breast; WS = White Stripping; SM = Spaghetti Meat) and different severities of myopathies (0 = absent, 1 = mild, 2 = severe) for cooking loss and average peak number from shear force curve on cooked fillets.

	Myopathies						
Parameter	NB	WB1 WS1	WB1 WS1 SM1	WB2 WS1	WB2 WS1 SM1	WB2 WS2*	WB2 WS2 SM1
Cooking loss(%)	15.30 ± 0.28^b^	15.58 ± 0.37^b^	18.49 ± 0.48^a^	20.05 ± 0.62^a^	18.39 ± 0.43^a^	18.49 ± 0.64^a^	19.07 ± 0.70^a^
Average Peak Number	2.90 ± 0.08^f^	3.47 ± 0.14^e^	4.37 ± 0.15^d^	6.09 ± 0.18^a^	4.98 ± 0.14^c^	6.04 ± 0.31^ab^	5.11 ± 0.15^bc^

a-d Means (N: NB = 85. WB1 WS1 = 37. WB1 WS1 SM1 = 31. WB2 WS1 = 44. WB2 WS1 SM1 = 61. WB2 WS2 = 4. WB2 WS2 SM1 = 25) ± SE followed by different superscript in a given row are significantly different (*P* < 0.05).

⁎ WB2 WS2 data was not used in the discussion due to low sample count.

The number of peaks in the shear force test has been suggested, by a few researches, as a possible way to identify myopathies (Morey and Owens, 2017; Bowker and Zhuang, 2019). Our results also show that WB2 fillets had the greatest number of peaks, with WB2 WS1 fillets having more than doubled the number of peaks as NB fillets (Table 2). The number of peaks was greater in SM breast fillets compared to the WB1 WS1 but not the WB2 WS1 fillets. Overall, peak count appears to be related to the number of connective tissue layers (i.e., replacing damaged muscle fibers). More work is needed to evaluate the relationship between peak count and sensory attributes of breast fillets.

## CONCLUSIONS

White Striping showed the least effect on breast fillets in all parameters measured. Because WS2 was only found in fillets when WB2 was also present (i.e., during our study), only values obtained from WB2 WS1 and WB2 WS2 were compared. Severe WS resulted in a higher pH, but no differences in compression force or average peak count compared to WS1. Mild Wooden Breast (WB1) without SM resulted in higher pH and compression force compared to NB fillets. This is in contrast to other studies which also reported significant changes in drip and cooking losses. The presence of severe WB (WB2) significantly increased drip loss, cooking loss, pH, compression force, peak count, and color values (L*, b*, and chroma) compared to the NB values. Samples with a combination up to SM1 could be evaluated whereas severe SM (SM 2) resulted in the complete separation of muscle fibers in breast fillets and made several tests impossible, including the determination and presence or severity of WS and WB. Therefore, only samples with a combination up to SM1 could be evaluated.

## References

[bib0001] Alnahhas N., Berri C., Chabault M., Chartrin P., Boulay M., Bourin M.C., Le Bihan-Duval E. (2016). Genetic parameters of white striping in relation to body weight, carcass composition, and meat quality traits in two broiler lines divergently selected for the ultimate pH of the pectoralis major muscle. BMC Genet.

[bib0002] Baldi G., Soglia F., Mazzoni M., Sirri F., Canonico L., Babini E., Laghi L., Cavani C., Petracci M. (2018). Implications of white striping and spaghetti meat abnormalities on meat quality and histological features in broilers. Animal.

[bib0003] Baldi G., Soglia F., Laghi L., Tappi S., Rocculi P., Tavaniello S., Prioriello D., Mucci R., Maiorano G., Petracci M. (2019). Comparison of quality traits among breast meat affected by current muscle abnormalities. Food Res. Int..

[bib38] Barbut S. (2015). *The Science of Poultry and Meat Processing*.

[bib0004] Barbut S. (2019). Recent myopathies in broiler's breast meat fillets. Worlds. Poult. Sci. J..

[bib0006] Bowker B., Zhuang H. (2019). Detection of razor shear force differences in broiler breast meat due to the woody breast condition depends on measurement technique and meat state. Poult. Sci..

[bib0007] Bowker B., Zhuang H. (2016). Impact of white striping on functionality attributes of broiler breast meat1. Poult. Sci..

[bib0008] Cai K., Shao W., Chen X., Campbell Y.L., Nair M.N., Suman S.P., Beach C.M., Guyton M.C., Schilling M.W. (2018). Meat quality traits and proteome profile of woody broiler breast (pectoralis major) meat. Poult. Sci..

[bib35] Cavitt L.C., Meullenet J.F., Gandhapuneni R.K., Youm G.W. (2005). Rigor development and meat quality of large and small broilers and the use of Allo-Kramer shear, needle puncture, and razor blade shear to measure texture. Poult. Sci..

[bib0009] Chatterjee D., Zhuang H., Bowker B.C., Rincon A.M., Sanchez-Brambila G. (2016). Instrumental texture characteristics of broiler pectoralis major with the wooden breast condition. Poult. Sci..

[bib0010] Che S., Wang C., Iverson M., Varga C., Barbut S., Bienzle D., Susta L. (2022). Characteristics of broiler chicken breast myopathies (spaghetti meat, woody breast, white striping) in Ontario, Canada. Poult. Sci..

[bib0011] Dalgaard L.B., Rasmussen M.K., Bertram H.C., Jensen J.A., Møller H.S., Aaslyng M.D., Hejbøl E.K., Pedersen J.R., Elsser-Gravesen D., Young J.F. (2018). Classification of wooden breast myopathy in chicken pectoralis major by a standardised method and association with conventional quality assessments. Int. J. Food Sci. Technol..

[bib0012] Dalle Zotte A., Tasoniero G., Puolanne E., Remignon H., Cecchinato M., Catelli E., Cullere M. (2017). Effect of “wooden breast” appearance on poultry meat quality, histological traits, and lesions characterization. Czech J. Anim. Sci..

[bib0013] Kuttappan V.A., Hargis B.M., Owens C.M. (2016). White striping and woody breast myopathies in the modern poultry industry: a review. Poult. Sci..

[bib0014] Kuttappan V.A., Owens C.M., Coon C., Hargis B.M., Vazquez-A Non M. (2017). Research Note Incidence of broiler breast myopathies at 2 different ages and its impact on selected raw meat quality parameters. Poult. Sci..

[bib0015] Lee Y.S., Owens C.M., Meullenet J.F. (2008). The meullenet-owens razor shear (mors) for predicting poultry meat tenderness: its applications and optimization. J. Texture Stud..

[bib0016] Lorenzi M., Mudalal S., Cavani C., Petracci M. (2014). Incidence of white striping under commercial conditions in medium and heavy broiler chickens in Italy. J. Appl. Poult. Res..

[bib0017] Malila Y., U-Chupaj J., Srimarut Y., Chaiwiwattrakul P., Uengwetwanit T., Arayamethakorn S., Punyapornwithaya V., Sansamur C., Kirschke C.P., Huang L., Tepaamorndech S., Petracci M., Rungrassamee W., Visessanguan W. (2018). Monitoring of white striping and wooden breast cases and impacts on quality of breast meat collected from commercial broilers (Gallus gallus). Asian-Australas. J. Anim. Sci..

[bib0018] Meullenet J.F., Jonville E., Grezes D., Owens C.M. (2004). Prediction of the texture of cooked poultry pectoralis major muscles by near-infrared reflectance analysis of raw meat. J. Texture Stud..

[bib0019] Morey A., Owens C.M. (2017). Poultry Quality Evaluation.

[bib0020] Mottet A., Tempio G. (2017). Global poultry production: current state and future outlook and challenges. Worlds. Poult. Sci. J..

[bib37] Mudalal S., Lorenzi M., Soglia F., Cavani C., Petracci M. (2015). Implications of white striping and wooden breast abnormalities on quality traits of raw and marinated chicken meat. Animal.

[bib0021] National Chicken Council. 2020. Statistics and research: broiler chicken industry key facts 2016.https://www.nationalchickencouncil.org/about-the-industry/statistics/broiler-chicken-industry-key-facts/, Accessed Nov. 2021.

[bib0022] Petracci M., Soglia F., Madruga M., Carvalho L., Ida E., Estévez M. (2019). Wooden-breast, white striping, and spaghetti meat: causes, consequences and consumer perception of emerging broiler meat abnormalities. Compr. Rev. Food Sci. Food Saf..

[bib0023] Sihvo H.K., Lindén J., Airas N., Immonen K., Valaja J., Puolanne E. (2017). Wooden breast myodegeneration of pectoralis major muscle over the growth period in broilers. Vet. Pathol..

[bib0025] Petracci M., Mudalal S., Bonfiglio A., Cavani C. (2013). Occurrence of white striping under commercial conditions and its impact on breast meat quality in broiler chickens. Poult. Sci..

[bib0024] Soglia F., Mazzoni M., Petracci M. (2019). Spotlight on avian pathology: current growth-related breast meat abnormalities in broilers. Avian Pathol.

[bib0026] Sun X., Koltes D.A., Coon C.N., Chen K., Owens C.M. (2018). Instrumental compression force and meat attribute changes in woody broiler breast fillets during short-term storage. Poult. Sci..

[bib0027] Tasoniero G., Bowker B., Stelzleni A., Zhuang H., Rigdon M., Thippareddi H. (2019). Use of blade tenderization to improve wooden breast meat texture. Poult. Sci..

[bib0028] Tasoniero G., Zhuang H., Gamble G.R., Bowker B.C. (2020). Effect of spaghetti meat abnormality on broiler chicken breast meat composition and technological quality. Poult. Sci..

[bib0029] Tijare V.V., Yang F.L., Kuttappan V.A., Alvarado C.Z., Coon C.N., Owens C.M. (2016). Meat quality of broiler breast fillets with white striping and woody breast muscle myopathies. Poult. Sci..

[bib34] Zhuang H., Savage E. (2013). Comparison of cook loss, shear force, and sensory descriptive profiles of boneless skinless white meat cooked from a frozen or thawed state. Poult. Sci..

[bib0030] Zuidhof M.J., Schneider B.L., Carney V.L., Korver D.R., Robinson F.E. (2014). Growth, efficiency, and yield of commercial broilers from 1957, 1978, and 20051. Poult. Sci..

